# Primary Cutaneous Mantle Cell Lymphoma: A Case Report

**DOI:** 10.1155/2013/394596

**Published:** 2013-05-23

**Authors:** L. D. Mazzuoccolo, G. A. Castro Perez, I. Sorin, A. I. Bravo

**Affiliations:** ^1^HIGA. Eva Perón, Gral San Martin, Buenos Aires, Argentina; ^2^Dermatology Unit, HIGA. Eva Perón, Gral San Martin, Buenos Aires, Argentina

## Abstract

Primary cutaneous mantle cell lymphoma (MCL) is a rare cutaneous proliferation of naive pregerminal CD-5 positive B cells in the skin with no extracutaneous involvement. Overexpression of cyclin D1 is pathognomonic of this condition, and surgery and radiation therapy are the most common therapeutic options. In this case, we describe the clinical, histopathological, immunohistochemical, and molecular characteristics of a new case of primary cutaneous MCL.

## 1. Introduction

Mantle cell lymphoma (MCL) is a clinical entity characterized by the proliferation of CD5-positive antigen-naïve pregerminal centre B cells within the mantle zone that surrounds normal germinal centre follicles [1]. This lymphoma is associated with the chromosomal translocation t(11, 14)(q13; q32) responsible for cyclin D1 overexpression [2–4].

Primary MCL is not included in the World Health Organization-European Organization for Research and Treatment of Cancer (WHO-EORTC) classification for cutaneous lymphomas [5].

## 2. Clinical Case 

**Figure 1:**
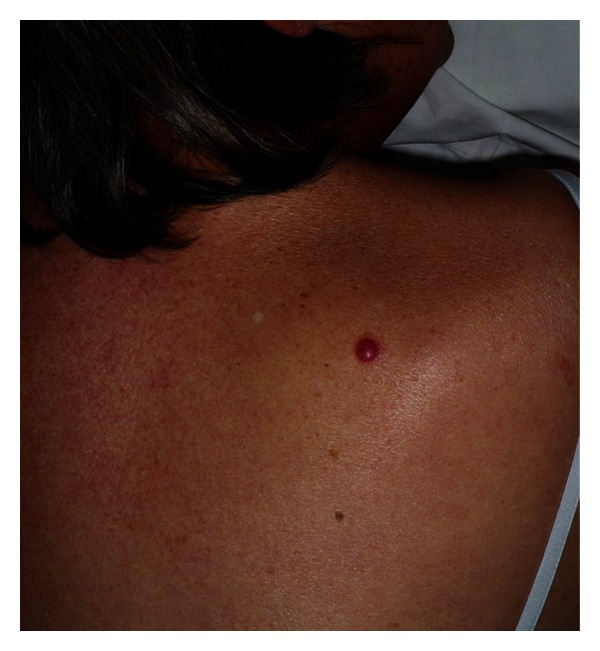
See the solitary and erythematous nodule of 1 cm in diameter on the patient's back.

A 72-year-old woman was seen in our clinic because of a solitary, slowly progressive erythematous nodule on the back of six months of duration. Her general health was otherwise unremarkable (Figure 1). 

**Figure 2:**
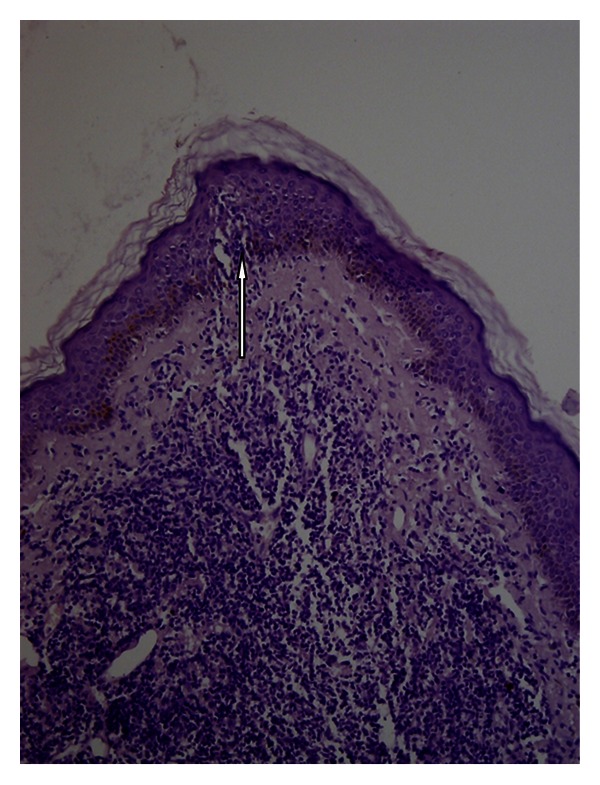
Histopathology: lymphoid dermal proliferation infiltrating epidermis (arrow) hematoxylin-eosin (10x).

Punch biopsy of the lesion was performed. Histopathological examination showed a diffuse lymphoid infiltrate of intermediate to large cells that involved the dermis (Figure 2). 

**Figure 3:**
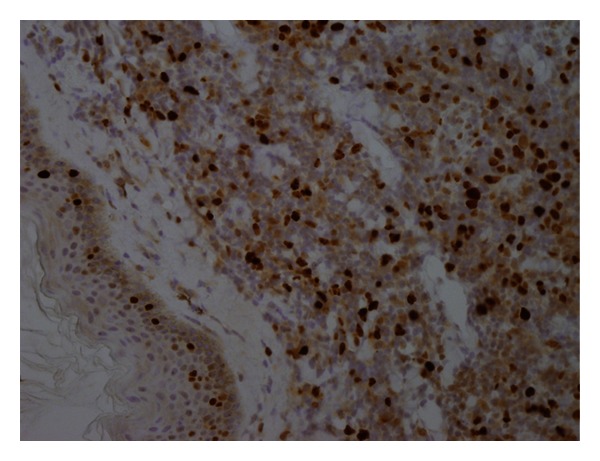
Positive cyclin D1 nuclear immunoreactivity in lymphoma cells (40x).

**Figure 4:**
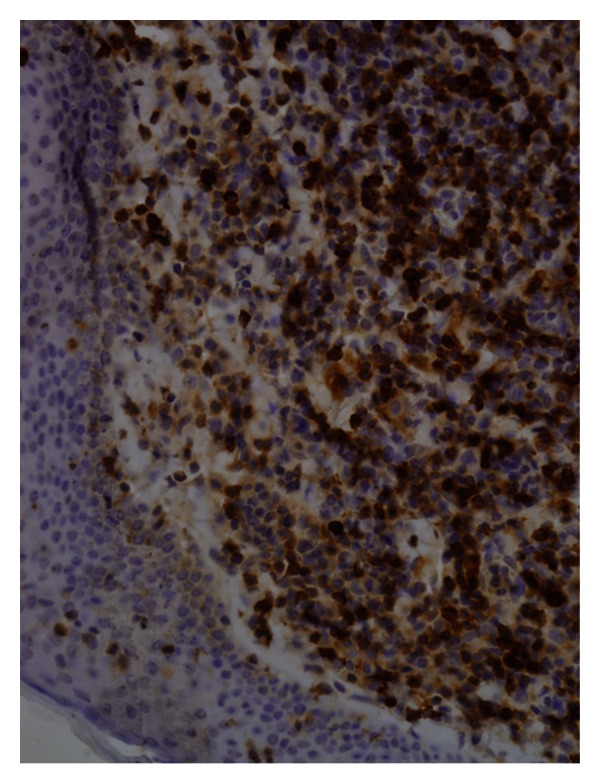
Positive CD5 immunoreactivity in cytoplasm of lymphoma cells (40x).

Immunohistochemistry showed the following phenotypical characteristics: CD3+, CD5+, CD20+, CD43+, CD45+, CD23−, BCL2+, and cyclin D1+ (Figures 3 and 4). On the basis of the histological and immunohistochemical data, a diagnosis of MCL was made.

Computed tomography scans of the head, neck, thorax, abdomen, and pelvis showed no pathological findings. Total body positron emission tomography (PET) did not reveal any systemic involvement.

Clinical staging showed no evidence of bone marrow or peripheral blood involvement. Histopathological flow cytometry and cytogenetic of the bone marrow revealed no evidence of MCL with karyotype 46 XX. A diagnosis of primary cutaneous MCL was therefore made and the nodule was operated.

**Figure 5:**
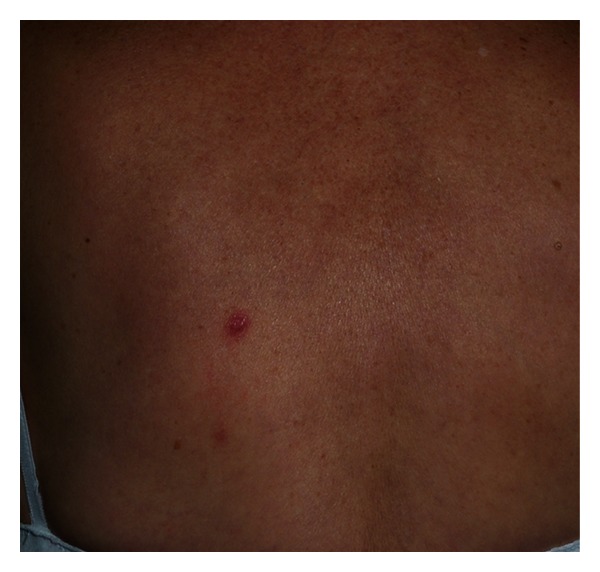
Second lesion. See similarity with the first nodule. Erythematous plaque of 0.7 cm in diameter.

After three months, a new lesion of similar characteristics was seen on the back, 15 cm away from the first. The new nodule was operated, and its result was the same (Figure 5). Clinical staging was negative again.

After 24 months of followup, no new lesions were seen.

## 3. Discussion

Secondary involvement of the skin in MCL has been described in several cases, whereas primary cutaneous MCLs have only exceptionally been reported [6, 7]. Skin involvement is described to occur in 17% of cases with stage IV MCL [8]. Patients who develop cutaneous disease with widespread MCL typically have a poor prognosis.

A primary cutaneous lymphoma, according to the WHO-EORTC definition [5], is a lymphoma that presents in the skin with no evidence of extracutaneous disease at the time of presentation. Our patient showed no signs of extracutaneous involvement at presentation or at 24 months after diagnosis. Clinical staging must include bone marrow biopsy.

The clinical features of the published cases are a 77-year-old man presented with several diffuse cutaneous erythematous nodules and plaques involving all body areas, a 78-year-old female presented with nodules on the breast area and on the back, a 76-year-old woman presented with a nodule on the thigh and an 83-year-old male with firm lesions on both thighs [6, 9–11].

Expression of cyclin D1 is pathognomonic for MCL and is an indirect evidence of translocation t(11 : 14) [2–4].

These patients without extracutaneous disease should not be treated with systemic chemotherapy. For this reason, it is fundamental to define whether it is a systemic lymphoma or a cutaneous one exclusively. Systemic MCL must be treated with chemotherapy, while the cutaneous one could be treated with local therapies such as surgery or radiotherapy. In our case, the patient was operated on two times, with no recurrences in each one.

Strict followup is necessary to detect new lesions or systemic involvement.

To the best of our knowledge, there have been only 4 patients reported who presented with a primary cutaneous MCL without evident systemic involvement, but the first was treated only with surgery.

As a conclusion, we can confirm that MCL can be observed primarily in the skin without systemic involvement and suggest that this entity should be introduced in upcoming classifications of cutaneous lymphomas. Regarding the management of primary cutaneous MCL—given the paucity of cases and the clinical behavior of primary cutaneous MCL—it is still not entirely clear, and guidelines regarding the management of such cases are lacking.
